# Wavier jet streams driven by zonally asymmetric surface thermal forcing

**DOI:** 10.1073/pnas.2200890119

**Published:** 2022-09-12

**Authors:** Woosok Moon, Baek-Min Kim, Gun-Hwan Yang, John S. Wettlaufer

**Affiliations:** ^a^Nordic Institute for Theoretical Physics, 106 91 Stockholm, Sweden;; ^b^Department of Environmental Atmospheric Sciences, Pukyong National University, 48513 Pusan, South Korea;; ^c^Department of Earth & Planetary Sciences, Yale University, New Haven, CT 06520;; ^d^Department of Mathematics, Yale University, New Haven, CT 06520;; ^e^Department of Physics, Yale University, New Haven, CT 06520

**Keywords:** wavier jet stream, Arctic amplification, planetary geostrophic motion, zonally-asymmetric thermal forcing

## Abstract

Recent studies have argued that global warming is responsible for a wavier jet stream, thereby driving midlatitude extreme flooding and drought. Polar amplification—the relative enhancement of high-latitude temperatures under global warming—is argued to be the principal climate state driving midlatitude extremes. Namely, the decreased meridional temperature gradient suppresses the mean zonal winds, leading to wavier midlatitude jets. However, although observations are consistent with such a linkage, a detailed dynamical mechanism is still debated. Here, we argue that the Northern Hemisphere land–sea thermal forcing contrast that underlies zonally asymmetric forcing drives a response in the planetary geostrophic motion, which provides balanced mean fields for synoptic eddies in midlatitudes and thus for wavier jet streams. We show that when the barotropic zonal mean wind *U* is smaller than a threshold, proportional to the *β*-plane effect and dry static stability, the flow field exhibits a dramatic transition from a response confined near the surface to one reaching the upper atmosphere. As global warming enhances polar amplification, the midlatitude jet stream intensity is suppressed. The confluence of these effects leads to wavier jet streams.

## Significance

Extreme midlatitude weather is strongly associated with the unusual meandering of jets. A central question is whether it is caused by or related to global warming. Here, we show that zonally asymmetric thermal forcing can drive a dramatic shift in planetary-scale atmospheric motion. As the zonal mean flow is reduced, a small-amplitude response confined near the surface shifts to a large-amplitude response reaching the upper atmosphere. As the high latitudes warm more rapidly under global warming, the reduction in zonal mean wind strength can trigger wavier atmospheric jets.

The connection between midlatitude jet structure and extreme weather events is a focus area in contemporary climate science (1–5). As westerly mean flows decrease, jets become wavier and quasi-stationary high- and low-pressure blocking patterns form, causing severe flooding and drought (see ref. 6 for a recent review). Although it is argued that Arctic amplification, wherein high latitudes experience enhanced warming, is responsible for wavier jets (7), there is neither a consensus on a central dynamical mechanism (8–12) nor a consensus on the climatology of blocking itself (6). Here we propose a dynamical linkage between the Arctic amplification and wavier midlatitude jets.

Baroclinic instability is generally considered to be the basic mechanism of generating the synoptic eddies that shape midlatitude weather patterns and control the magnitude of the poleward heat flux (13). Essential is the relationship between the meridional temperature gradient and the vertical shear of the zonal flow; when the vertical shear exceeds a critical value, associated with the upper-level westerly flow, the instability can be triggered (14, 15). On the other hand, Arctic amplification is a hemispheric-scale phenomenon providing the conditions for the development of large-scale eddies in midlatitudes, wherein the vertical shear of the zonal mean winds is weakened. Under such circumstances, the frequency and intensity of baroclinic instability are expected to decrease, making it unlikely to underlie wavier jets.

Meridional fluctuations of midlatitude jets occur on much larger spatial scales and longer temporal scales than synoptic eddies (16, 17). In particular, the spatial scale is larger than the external Rossby deformation radius of 3,000 km and close to Earth’s radius. The temporal scales are longer than that of weather, but shorter than seasonal. Therefore, quasi-geostrophic potential vorticity cannot explain planetary-scale jet dynamics. Indeed, theory shows that there exist planetary-scale motions with spatiotemporal scales that are larger than the quasi-geostrophic atmospheric motions (18–20). Whereas vorticity dynamics underlie synoptic-scale motions, heat flux balance underlies planetary-scale motions (19). For example, by parameterizing the synoptic-scale poleward heat flux, one can derive a planetary-scale heat equation (21). In contrast to canonical baroclinic instability, on the planetary scale the instability is triggered when the vertical shear of the jet is below a threshold, with the most unstable waves being nearly stationary or weakly westward propagating modes. Therefore, as noted above, this is why the conditions favorable for Arctic amplification are also favorable for planetary-scale baroclinic waves, which arise from the combination of vertical shear and the Sverdrup relation, which relates the vertical velocity to the meridional velocity due to the planetary beta effect.

The discussion thus far deals solely with zonally symmetric conditions and hence ignores asymmetric processes such as land–ocean thermal contrast or orographic forcing. Thus, from within a multiscale framework (19), whereas zonally asymmetric thermal forcing drives meridional and vertical velocities on the planetary scale, planetary-scale geostrophic motion provides a mean field for the development of synoptic-scale eddies. In quasi-geostrophic dynamics the vertical propagation of waves depends on the zonal mean wind and the zonal wavelength; as the former decreases the latter increases and waves generated from zonally asymmetric surface forcing can propagate upward into the stratosphere (22). Thus, because planetary geostrophic motion is a limit of the quasi-geostrophic vorticity equation, the meridional displacement in response to zonally asymmetric thermal forcing in these two limits will be different. Through this lens we examine the dynamics of jets under the influence of global warming. The central issues are described presently.

Within the framework of planetary geostrophic motion we consider the barotropic mean field defined by the zonal mean wind. A linearized treatment leads to an inhomogeneous heat transport equation with spatially varying thermal forcing and the Sverdrup relation, arising from the continuity equation. For given boundary conditions, these two equations produce the magnitude and spatial patterns of the response to the thermal forcing. This provides the balanced background field for the vorticity dynamics of synoptic eddies. The approach provides a mechanism linking wavier midlatitude jets with weakened mean flow induced by Arctic amplification. Finally, we find the mechanism to be operative in simulations of an idealized global climate model based on the primitive equations.

## Planetary Geostrophic Motion

The general large-scale dynamics of the atmosphere can be framed in terms of eddy–mean-flow interactions (23). Synoptic-scale midlatitude baroclinic instability and meridional temperature gradients drive shear instabilities that generate synoptic-scale eddies. These eddies grow by extracting energy from mean fields, beginning their life cycle near the surface and eventually dissipating in the upper atmosphere (24). The life cycle of baroclinic eddies is driven by wave–mean-field interactions, acting as an engine that transfers residual heat in low latitudes to the polar regions, thereby controlling the low-frequency dynamics of the large-scale midlatitude atmosphere (25).

Although studies of eddy–mean-flow interactions begin through identification of the mean-field flow, this identification is not prescriptive. However, a necessary condition is that the characteristic time and length scales of the mean field must be much larger than those of the eddies. In the commonly treated zonally symmetric atmosphere, the mean is normally the zonally averaged field. Hence, when thermally forced in a zonally symmetric manner, the resulting statistical flow field characterized by synoptic eddies should be zonally symmetric to produce an appropriate zonally-averaged mean field (26). Although it is commonly assumed that the asymmetric contribution is small (27), here we quantitatively address the consequences of zonally asymmetric forcing associated with land–ocean contrasts.

When scales increase from synoptic to planetary, Burger (28) recognized the existence of two types of geostrophic motion and Phillips (29) formulated what are now commonly called the planetary geostrophic equations. Whereas on length scales L~1000 km, the equation of motion is the quasi-geostrophic vorticity equation, on length scales of approximately the radius of Earth, the leading-order dynamics are governed by the planetary geostrophic equations (18, 19). One can treat the overall dynamics of the large-scale atmosphere through the mutual interaction of these two scales (19, 30).

On the planetary scale, the key process is the energy flux balance, wherein the poleward heat flux is controlled by turbulent synoptic eddies. On the synoptic scale, the key process is conservation of potential vorticity, wherein the planetary-scale wind provides the mean field for synoptic motions. In this multiscale framework, planetary geostrophic motion provides the mean field and its temporal and spatial scales are asymptotically larger than those of the synoptic scales. Thus, the planetary-scale wind is the background field in the quasi-geostrophic potential vorticity equation (19). Therefore, the baroclinic stability of planetary geostrophic motion determines the initial growth of synoptic eddies and their life cycle. This framework for planetary dynamics allows us to examine the effect of zonally asymmetric thermal forcing on the jet stream. Although the thermal forcing influences both scales, it is essential to examine the planetary dynamics, because, as described next, synoptic eddies are generated by the stability of the balanced planetary geostrophic motion.

## Linearized Planetary Geostrophic Motion

When we neglect the interaction with synoptic eddies, the dimensionless form of planetary geostrophic motion is given by

[1] $$u_{L}=-\frac{\partial P_{L}}{\partial y},\;\;v_{L}=\frac{\partial P_{L}}{\partial x},\;\;\Theta_{L}=\frac{\partial P_{L}}{\partial z},$$

[2] $$\frac{1}{\rho_{s}}\frac{\partial }{\partial z}(\rho_{s}w_{L})-\beta_{L}v_{L}=0\;\;\text{and}$$

[3] $$\frac{\partial \Theta_{L}}{\partial t}+u_{L}\frac{\partial \Theta_{L}}{\partial x}+v_{L}\frac{\partial \Theta_{L}}{\partial y}+w_{L}\left(S+\frac{\partial \Theta_{L}}{\partial z}\right)=Q_{L},$$

where *u_L_*, *v_L_*, and *w_L_* are the velocities in the *x*, *y*, and *z* directions, respectively; *P_L_* is the pressure; and Θ*_L_* is the potential temperature, with *S* the dimensionless vertical stability (19). The subscript *L* denotes the planetary scale, or approximately wavenumbers 1 to 4 in the Fourier domain (19). The continuity equation contains the average density profile, $\rho_{s}=\rho_{s}(z)$, which leads to the Sverdrup relationship, Eq. **2**, connecting the vertical velocity *w_L_* to the meridional velocity *v_L_* associated with the planetary *β*-effect. The heat transport Eq. **3** represents the spatiotemporal evolution of the potential temperature for a given thermal forcing, *Q_L_*, which may in general depend on space and time.

Assume that the dominant mean flow is a zonal mean barotropic wind, *U*, driven by the mean pressure $P_{L}^{S}=-Uy$. Let the total pressure field be $P_{L}=P_{L}^{S}+\phi_{L}$, where $\phi_{L}$ is a zonally asymmetric pressure perturbation satisfying $\phi_{L}\ll P_{L}^{S}$, generated by weak thermal forcing *Q_L_*. Now the linearized steady-state planetary geostrophic equations are

[4] $$U\frac{\partial }{\partial x}\frac{\partial \phi_{L}}{\partial z}+Sw_{L}=Q_{L}(x,z)\equiv Q_{0}G(x)e^{-k_{s}z}$$

[5] $$\left(\frac{\partial }{\partial z}-\frac{1}{H}\right)w_{L}=\beta_{L}\frac{\partial \phi_{L}}{\partial x},$$

where $\frac{1}{\rho_{s}}\frac{\partial \rho_{s}}{\partial z}=-\frac{1}{H}$ with *H* a constant scale height. The zonally asymmetric thermal forcing $Q_{L}(x,z)$ has amplitude *Q*_0_ and $e^{-k_{s}z}$ treats the thermal forcing adjacent to the surface.

Combining Eqs. **4** and **5** leads to

[6] $$\begin{array}{l} U\frac{\partial }{\partial x}\frac{\partial }{\partial z}\left(\frac{\partial }{\partial z}-\frac{1}{H}\right)\phi_{L}+\beta_{L}S\frac{\partial \phi_{L}}{\partial x}\\ \;=-\left(k_{s}+\frac{1}{H}\right)Q_{0}G(x)e^{-k_{s}z}, \end{array}$$

from which, upon zonal integration, the central result follows as

[7] $$\frac{\partial^{2}\phi_{L}}{\partial z^{2}}-\frac{1}{H}\frac{\partial \phi_{L}}{\partial z}+\frac{\beta_{L}S}{U}\phi_{L}=-\left(k_{s}+\frac{1}{H}\right)\frac{Q_{0}}{U}F(x)e^{-k_{s}z},$$

where F′(x)=G(x). Clearly the solutions of this forced second-order equation depend on the boundary conditions for the vertical velocity *w_L_*. We consider two cases: 1) *w_L_* = 0 at the surface (*z* = 0) and at an upper level (*z* = 1), treating the tropopause as a rigid boundary, and 2) *w_L_* = 0 at the surface (*z* = 0) and $\lim_{z\to \infty }\rho_{s}w_{L}<\infty$ and $\lim_{z\to \infty }\rho_{s}\bar{w_{L}\phi_{L}}>0$, implying the upward propagation of energy flux due to the surface thermal forcing. Here, $\bar{M}$ denotes the horizontal average of *M* (31).

## Results

The general solution of the inhomogeneous Eq. **7** is $\phi_{L}=\phi_{L}^{H}+\phi_{L}^{P}$, where the homogeneous part, $\phi_{L}^{H}$, describes the internal modes and the particular part, $\phi_{L}^{P}$, describes the direct response to the forcing.

The central results of our analysis are characterized by the magnitude of the zonal mean wind, *U*, relative to a threshold, $U_{\text{th}}\equiv 4H^{2}\beta_{L}S$, which captures the strength of the vertical stability and the *β*-effect. Independent of the boundary conditions, when $U<U_{\text{th}}$ ($U>U_{\text{th}}$), the response of the system is sinusoidal (exponentially decaying). Therefore, as the vertical stability increases with *S* and *H*, so too does $U_{\text{th}}$ and the internal modes of the system reflect the propagation of surface forcing and hence wavier jet streams. We now demonstrate this and explore the detailed dependence of the modes of the system on the boundary conditions and the principal physical parameters.

### A. Two Rigid Boundaries.

We approximate the tropopause, the boundary between the troposphere and the stratosphere, as a “rigid lid” by imposing $w_{L}(z=0)$ = $w_{L}(z=1)=0$, where the surface is *z* = 0 and the tropopause is *z* = 1. The homogeneous solution, $\phi_{L}^{H}$, is determined by the characteristic equation, $x^{2}-\frac{1}{H}x+\beta_{L}S/U=0$, and hence the sign of the discriminant $\frac{1}{4H^{2}}-\frac{\beta_{L}S}{U}$ determines its nature.

#### A.1. $U>4H^{2}\beta_{L}S$.

The solution of Eq. **7** is

[8] $$\begin{array}{l} \phi_{L}=\phi_{L}^{H}+\phi_{L}^{P}\\ \equiv Ae^{\left(\frac{1}{2H}+q\right)z}+Be^{\left(\frac{1}{2H}-q\right)z}\\ -\frac{Q_{0}\left(k_{s}+\frac{1}{H}\right)F(x)}{U\left(k_{s}^{2}+\frac{1}{H}k_{s}\right)+\beta_{L}S}e^{-k_{s}z}, \end{array}$$

where $q=\sqrt{\frac{1}{4H^{2}}-\frac{\beta_{L}S}{U}}$ and *A* and *B* are the undetermined coefficients of $\phi_{L}^{H}$. The vertical velocity *w_L_* from Eq. **4** is

[9] $$w_{L}=-\frac{U}{S}\frac{\partial }{\partial x}\frac{\partial \phi_{L}}{\partial z}+\frac{Q_{0}}{S}G(x)e^{-k_{s}z}.$$

The boundary condition at the surface, $w_{L}(z=0)=0$, gives

[10] $$\begin{array}{l} \frac{\partial A}{\partial x}\left(\frac{1}{2H}+q\right)+\frac{\partial B}{\partial x}\left(\frac{1}{2H}-q\right)\\ \;=\frac{Q_{0}}{U}G(x)\frac{\beta_{L}S}{U\left(k_{s}^{2}+\frac{1}{H}k_{s}\right)+\beta_{L}S}, \end{array}$$

and that at the tropopause, $w_{L}(z=1)=0$, leads to

[11] $$\begin{array}{l} \frac{\partial A}{\partial x}\left(\frac{1}{2H}+q\right)e^{\left(\frac{1}{2H}+q\right)}+\frac{\partial B}{\partial x}\left(\frac{1}{2H}-q\right)e^{\left(\frac{1}{2H}-q\right)}\\ \;=\frac{Q_{0}}{U}G(x)e^{-k_{s}}\frac{\beta_{L}S}{U\left(k_{s}^{2}+\frac{1}{H}k_{s}\right)+\beta_{L}S}, \end{array}$$

which combined determine the coefficients *A* and *B* as

[12] $$\begin{array}{l} A=\frac{Q_{0}F(x)}{2e^{\frac{1}{2H}}\text{sinh}\;q}\frac{\frac{1}{2H}-q}{U\left(k_{s}^{2}+\frac{1}{H}k_{s}\right)+\beta_{L}S}\left(-e^{\frac{1}{2H}-q}+e^{-k_{s}}\right)\text{and}\\ B=\frac{Q_{0}F(x)}{2e^{\frac{1}{2H}}\text{sinh}\;q}\frac{\frac{1}{2H}+q}{U\left(k_{s}^{2}+\frac{1}{H}k_{s}\right)+\beta_{L}S}\left(e^{\frac{1}{2H}+q}-e^{-k_{s}}\right). \end{array}$$

Thus, armed with these coefficients we obtain the solutions

[13] $$\begin{array}{l} \phi_{L}=\;\frac{Q_{0}F(x)}{U\left(k_{s}^{2}+\frac{1}{H}k_{s}\right)+\beta_{L}S}[-\frac{e^{\frac{1}{2H}-q}-e^{-k_{s}}}{2e^{\frac{1}{2H}}\text{sinh}\;q}e^{\left(\frac{1}{2H}+q\right)z}\\ +\frac{e^{\frac{1}{2H}+q}-e^{-k_{s}}}{2e^{\frac{1}{2H}}\text{sinh}\;q}e^{\left(\frac{1}{2H}-q\right)z}-\left(k_{s}+\frac{1}{H}\right)e^{-k_{s}z}] \end{array}$$

and

[14] $$\begin{array}{l} w_{L}=\;\frac{\beta_{L}Q_{0}G(x)}{U\left(k_{s}^{2}+\frac{1}{H}k_{s}\right)+\beta_{L}S}[\frac{e^{\frac{1}{2H}-q}-e^{-k_{s}}}{2e^{\frac{1}{2H}}\text{sinh}\;q}e^{\left(\frac{1}{2H}+q\right)z}\\ -\frac{e^{\frac{1}{2H}+q}-e^{-k_{s}}}{2e^{\frac{1}{2H}}\text{sinh}\;q}e^{\left(\frac{1}{2H}-q\right)z}-\left(k_{s}+\frac{1}{H}\right)e^{-k_{s}z}]. \end{array}$$

**Fig. 1.**
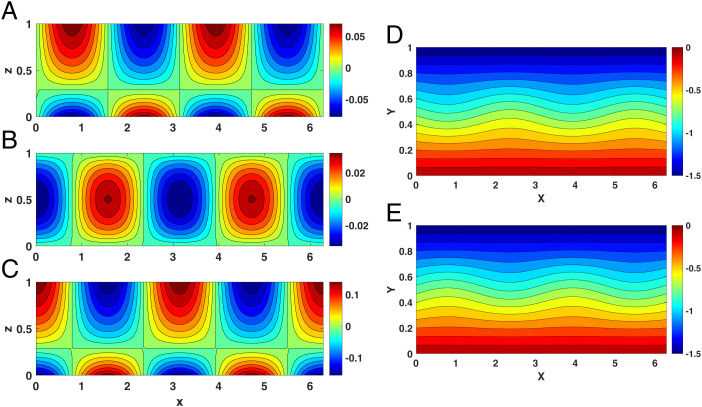
The response of planetary geostrophic motion to thermal forcing when $U>4H^{2}\beta_{L}S$, with G(x)=cos(2x), F(x)=sin(2x)/2, *k_s_* = 2, *H* = 0.5, $\beta_{L}=1.0$, *S* = 1.0, and *U* = 1.5. (*A–E*) The pressure field, $\phi_{L}$ (*A*); the vertical wind, *w_L_* (*B*); the meridional wind, *v_L_* (*C*); and the total pressure field $P_{L}=-Uy+\phi_{L}$ at (*D*) the surface (*z* = 0) and (*E*) the top of atmosphere (*z* = 1). Note that the flow is confined to a surface region less than approximately *H*.

When $U>U_{\text{th}}$, the internal modes represented by the homogeneous solution have much smaller magnitude than the mean wind *U*. Fig. 1 shows an example of this case where G(x)=cos(2x) and F(x)=sin(2x)/2 represent the zonal distribution of the thermal forcing, noting that the land–ocean contrast in the Northern Hemisphere exhibits a wavenumber 2 structure. The pressure field, $\phi_{L}$, is proportional to *F*(*x*), but the winds, *w_L_* and *v_L_*, are proportional to *G*(*x*), which leads to the observed π/2 phase difference between them; compare Fig. 1*A* with Fig. 1 *B* and *C*.

In the large-scale atmosphere the horizontal velocity field is parallel to the isobars, allowing us to see the effect of the thermal forcing on the horizontal velocity field by plotting the total pressure, $P_{L}=-Uy+\phi_{L}$, at the surface (*z* = 0) and the tropopause (*z* = 1), in Fig. 1 *D* and *E*, respectively. The surface thermal forcing does not generate a significant zonally asymmetric response, and hence the mean wind field is almost zonally symmetric.

#### A.2. $U<4H^{2}\beta_{L}S$.

As *U* decreases below the threshold $4H^{2}\beta_{L}S$, the characteristic equation for the homogeneous solution has two real and two complex solutions, so that the internal modes will exhibit a different response to the thermal forcing than in *Section A.1*.

In this case, the solution of Eq. **7** is

[15] $$\begin{array}{l} \phi_{L}=\;Ae^{\left(\frac{1}{2H}+im\right)z}+Be^{\left(\frac{1}{2H}-im\right)z}\\ -\frac{Q_{0}\left(k_{s}+\frac{1}{H}\right)F(x)}{U\left(k_{s}^{2}+\frac{1}{H}k_{s}\right)+\beta_{L}S}e^{-k_{s}z}, \end{array}$$

where $m=\sqrt{\frac{\beta_{L}S}{U}-\frac{1}{4H^{2}}}$, and *A* and *B* are coefficients to be determined by the two boundary conditions. Using Eq. **4** the vertical velocity *w_L_* is

[16] $$\begin{array}{l} w_{L}=\;-\sqrt{\frac{\beta_{L}U}{S}}e^{\frac{z}{2H}}\left[\frac{\partial A}{\partial x}e^{i(mz+\psi )}+\frac{\partial B}{\partial x}e^{-i(mz+\psi )}\right]\\ +\frac{\beta_{L}Q_{0}G(x)}{U\left(k_{s}^{2}+\frac{1}{H}k_{s}\right)+\beta_{L}S}e^{-k_{s}z}. \end{array}$$

Applying the boundary conditions, $w_{L}(z=0)=w_{L}(z=1)=0$, leads to

[17] $$\frac{\partial A}{\partial x}e^{i\psi }+\frac{\partial B}{\partial x}e^{-i\psi }=\sqrt{\frac{\beta_{L}S}{U}}\frac{Q_{0}G(x)}{U\left(k_{s}^{2}+\frac{1}{H}k_{s}\right)+\beta_{L}S}$$

and

[18] $$\begin{array}{l} \frac{\partial A}{\partial x}e^{i(m+\psi )}+\frac{\partial B}{\partial x}e^{-i(m+\psi )}\\ \;=\sqrt{\frac{\beta_{L}S}{U}}\frac{Q_{0}G(x)}{U\left(k_{s}^{2}+\frac{1}{H}k_{s}\right)+\beta_{L}S}e^{-\left(k_{s}+\frac{1}{2H}\right)}. \end{array}$$

Combining Eqs. **17** and **18**, we obtain

[19] $$\begin{array}{l} \frac{\partial A}{\partial x}=\;\frac{\sqrt{\beta_{L}S}}{\sqrt{U}}\frac{Q_{0}G(x)}{U\left(k_{s}^{2}+\frac{1}{H}k_{s}\right)+\beta_{L}S}\\ \times \frac{1}{2i\text{sin}(m)}\left[-e^{-i(m+\psi )}+e^{-\left(k_{s}+\frac{1}{2H}\right)-i\psi }\right] \end{array}$$

and

[20] $$\begin{array}{l} \frac{\partial B}{\partial x}=\;\frac{\sqrt{\beta_{L}S}}{\sqrt{U}}\frac{Q_{0}G(x)}{U\left(k_{s}^{2}+\frac{1}{H}k_{s}\right)+\beta_{L}S}\\ \times \frac{1}{2i\text{sin}(m)}\left[e^{i(m+\psi )}-e^{-\left(k_{s}+\frac{1}{2H}\right)+i\psi }\right], \end{array}$$

from which the solutions follow as

[21] $$\begin{array}{l} \phi_{L}=\frac{Q_{0}F(x)}{U\left(k_{s}^{2}+\frac{1}{H}k_{s}\right)+\beta_{L}S}\\ \;\times \frac{\sqrt{\beta_{L}S}}{\sqrt{U}}e^{\frac{z}{2H}}\left[\frac{-\text{sin}(mz-m-\psi )+e^{-\left(k_{s}+\frac{1}{2H}\right)}\text{sin}(mz-\psi )}{\text{sin}(m)}\right]\\ \;-\frac{Q_{0}\left(k_{s}+\frac{1}{H}\right)F(x)}{U\left(k_{s}^{2}+\frac{1}{H}k_{s}\right)+\beta_{L}S}e^{-k_{s}z} \end{array}$$

and

[22] $$\begin{array}{l} w_{L}=\frac{\beta_{L}Q_{0}G(x)}{U\left(k_{s}^{2}+\frac{1}{H}k_{s}\right)+\beta_{L}S}\\ \;\times e^{\frac{z}{2H}}\left[\frac{\text{sin}(mz-m)-e^{-\left(k_{s}+\frac{1}{2H}\right)}\text{sin}(mz)}{\text{sin}(m)}\right]\\ \;+\frac{\beta_{L}Q_{0}G(x)}{U\left(k_{s}^{2}+\frac{1}{H}k_{s}\right)+\beta_{L}S}e^{-k_{s}z}. \end{array}$$

**Fig. 2.**
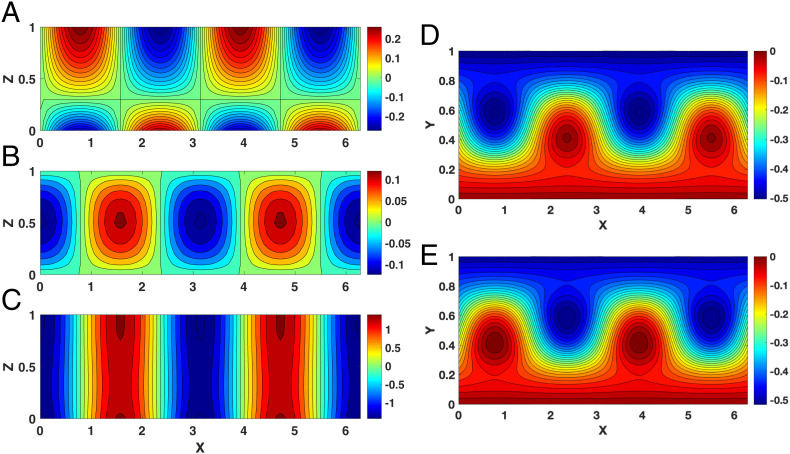
The response of planetary geostrophic motion to surface thermal forcing when $U<4H^{2}\beta_{L}S$. Here *U* = 0.5 and the other parameters are the same as in Fig. 1. (*A–E*) The pressure field, $\phi_{L}$ (*A*); vertical wind, *w_L_* (*B*); meridional wind, *v_L_* (*C*); and the pressure field $P_{L}=-Uy+\phi_{L}$ at (*D*) the surface (*z* = 0) and (*E*) the top of atmosphere (*z* = 1).

Fig. 2, where we plot the $\phi_{L}$, *w_L_*, and *v_L_* fields in the *x* – *z* plane, clearly shows that when the mean zonal velocity, *U*, is smaller than the threshold, $4H^{2}\beta_{L}S$, the nature of the solutions changes dramatically. In contrast to the case when $U>4H^{2}\beta_{L}S$, the atmospheric response to the thermal forcing is nonnegligible, reaching the top of atmosphere in the pressure and meridional velocity fields, and continuity leads to a maximum vertical velocity in the middle of atmosphere.

The behavior below and above threshold is best compared through the total pressure, $P_{L}=-Uy+\phi_{L}$ (Figs. 1 *D* and *E* and 2 *D* and *E*). Clearly, the magnitude of the response is larger and wavier in the latter.

### B. Unbounded Upper Domain (z=∞).

The tropopause confounds the problem of rigorously specifying an upper boundary condition; we can rationalize a rigid lid, as in *Sections A.1* and *A.2*, a free boundary for the large-scale tropospheric flow, or treat the atmosphere as semi-infinite. Here we impose boundary conditions in the far field (z=∞) such that $\rho_{s}|\phi_{L}{|}^{2}<\infty$ and $\rho_{s}\bar{\phi_{L}w_{L}}>0$ (31), the latter condition implying an upward propagation of signals from the surface, but it precludes downward propagation. Beyond this, the magnitude of *U* relative to $4H^{2}\beta_{L}S$ determines the overall nature of vertical propagation, as described next.

#### B.1. $U>4H^{2}\beta_{L}S$.

The solution in this case is

[23] $$\phi_{L}=Be^{\left(\frac{1}{2H}-q\right)z}-\frac{Q_{0}\left(k_{s}+\frac{1}{H}\right)F(x)}{U\left(k_{s}^{2}+\frac{1}{H}k_{s}\right)+\beta_{L}S}e^{-k_{s}z},$$

where the term $e^{\left(\frac{1}{2H}+q\right)z}$ is discarded since it violates the boundary condition $\rho_{s}|\phi_{L}{|}^{2}<\infty$. We use Eq. **4** to find

[24] $$\begin{array}{l} w_{L}=\;-\frac{U}{S}\frac{\partial B}{\partial x}\left(\frac{1}{2H}-q\right)e^{\left(\frac{1}{2H}-q\right)z}\\ +\frac{\beta_{L}Q_{0}G(x)}{U\left(k_{s}^{2}+\frac{1}{H}k_{s}\right)+\beta_{L}S}e^{-k_{s}z}, \end{array}$$

from which we determine *B* using the surface boundary condition, $w_{L}(z=0)=0$, as

[25] $$B=\frac{Q_{0}F(x)}{U\left(\frac{1}{2H}-q\right)}\frac{\beta_{L}S}{U\left(k_{s}^{2}+\frac{1}{H}k_{s}\right)+\beta_{L}S}.$$

The pressure and vertical velocity are thus found to be

[26] $$\begin{array}{l} \phi_{L}=\;\frac{Q_{0}F(x)}{U\left(k_{s}^{2}+\frac{1}{H}k_{s}\right)+\beta_{L}S}\\ \times \left[\frac{\beta_{L}S}{U\left(\frac{1}{2H}-q\right)}e^{\left(\frac{1}{2H}-q\right)z}-\left(k_{s}+\frac{1}{H}\right)e^{-k_{s}z}\right] \end{array}$$

and

[27] $$w_{L}=\frac{\beta_{L}Q_{0}G(x)}{U\left(k_{s}^{2}+\frac{1}{H}k_{s}\right)+\beta_{L}S}\left[e^{-k_{s}z}-e^{\left(\frac{1}{2H}-q\right)z}\right].$$

**Fig. 3.**
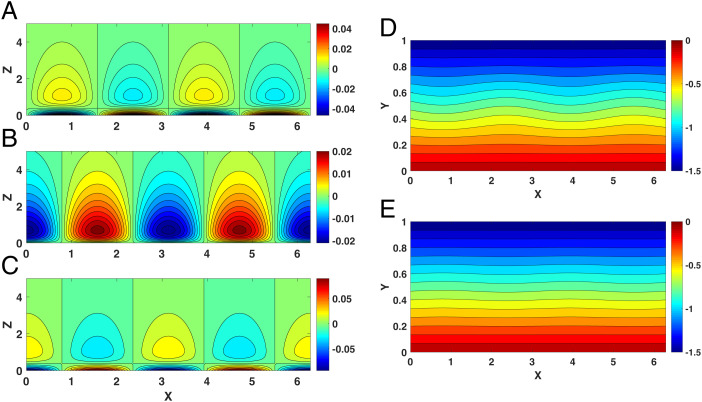
The response of planetary geostrophic motion to thermal forcing confined near the surface when $U>4H^{2}\beta_{L}S$ and *U* = 1.5, with the other parameters the same as in Fig. 1, but note the difference in the vertical scale. (*A–E*) The pressure field, $\phi_{L}$ (*A*); vertical wind, *w_L_* (*B*); meridional wind, *v_L_* (*C*); and the total pressure field $P_{L}=-Uy+\phi_{L}$ at (*D*) the surface (*z* = 0) and (*E*) the tropopause (*z* = 1).

Similar to the rigid lid case, for $U>U_{\text{th}}$ the response is smaller and surface confined, as shown in the pressure and velocity fields plotted in Fig. 3, in which all the parameters are the same as in Fig. 1. Note that we multiply all field variables by $e^{-\frac{1}{2H}z}$ to compensate for the exponential factor emerging from the mean density profile $\rho_{s}(z)$. Clearly, a strong zonal mean westerly wind suppresses the propagation of the response. Moreover, this is reflected in the vertical decay of the pressure field shown in Fig. 3 *D* and *E*, which is wavier at the surface but nearly zonally symmetric (– *Uy*) aloft, a typical mean atmospheric field.

#### B.2. $U<4H^{2}\beta_{L}S$.

In this regime the two homogeneous solutions, or internal modes, are proportional to $e^{\frac{1}{2H}\pm im}$, where $m=\sqrt{\frac{\beta_{L}S}{U}-\frac{1}{4H^{2}}}$. Although both solutions satisfy the condition $\rho_{s}|\phi_{L}{|}^{2}<\infty$, only one satisfies the far-field boundary condition at z=∞. Thus, appealing to the condition that $\rho_{s}\bar{\phi_{L}w_{L}}>0$, the only possible mode propagates upward, viz., $e^{\frac{1}{2H}+im}$ (31).

These considerations lead to the solution for $\phi_{L}$ as

[28] $$\phi_{L}=Ae^{\left(\frac{1}{2H}+im\right)z}-\frac{Q_{0}\left(k_{s}+\frac{1}{H}\right)F(x)}{U\left(k_{s}^{2}+\frac{1}{H}k_{s}\right)+\beta_{L}S}e^{-k_{s}z},$$

and imposing the boundary condition at the surface, $w_{L}(z=0)=0$, provides *A* as

[29] $$A=\frac{Q_{0}G(x)}{U\left(\frac{1}{2H}+im\right)}\frac{\beta_{L}S}{U\left(k_{s}^{2}+\frac{1}{H}k_{s}\right)+\beta_{L}S},$$

thereby giving

[30] $$\begin{array}{l} \phi_{L}=\;\frac{Q_{0}F(x)}{U\left(k_{s}^{2}+\frac{1}{H}k_{s}\right)+\beta_{L}S}\\ \times \left[e^{\frac{z}{2H}}\sqrt{\frac{\beta_{L}S}{U}}\text{cos}(mz-\psi )-\left(k_{s}+\frac{1}{H}\right)e^{-k_{s}z}\right] \end{array}$$

and

[31] $$w_{L}=\frac{Q_{0}\beta_{L}G(x)}{U\left(k_{s}^{2}+\frac{1}{H}k_{s}\right)+\beta_{L}S}\left[e^{-k_{s}z}-e^{\frac{1}{2H}z}\text{cos}(mz)\right].$$

**Fig. 4.**
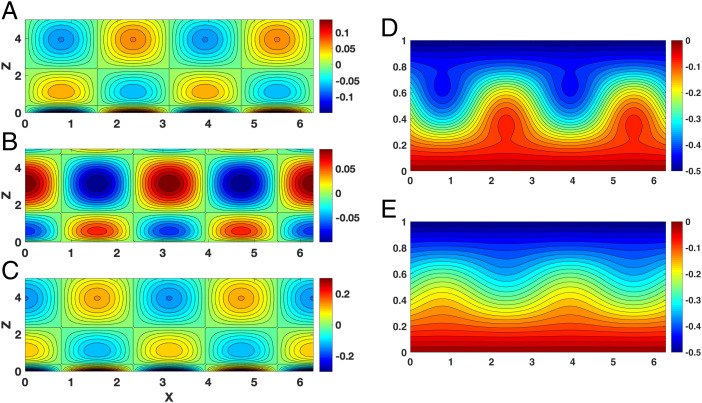
The response of planetary geostrophic motion to surface thermal forcing when $U<4H^{2}\beta_{L}S$. Here *U* = 0.5 and the other parameters are the same as in Fig. 1. (*A–E*) The pressure field, $\phi_{L}$ (*A*); vertical wind, *w_L_* (*B*); meridional wind, *v_L_* (*C*); and the total pressure field $P_{L}=-Uy+\phi_{L}$ at the (*D*) surface (*z* = 0) and (*E*) top of the atmosphere (*z* = 1).

Now, in contrast to Fig. 3, as the mean westerly flow weakens so that $U<U_{\text{th}}$, confinement is replaced by propagating wave behavior. Fig. 4 shows that all of the field variables exhibit a wave pattern in the vertical with westward phase shifts; the pressure has a π/2 phase shift relative to the vertical and meridional winds. Thus, while the surface thermal forcing is maximal near the surface, it propagates vertically and westward. This is further reflected in the breaking of zonal symmetry of the total pressure field throughout the troposphere as shown in Fig. 4 *D* and *E*. Our central finding is that the weakening of the midlatitude barotropic mean wind relative to the stability-dependent threshold $U_{\text{th}}=4H^{2}\beta_{L}S$ controls the vertical propagation of the surface thermal forcing and thus the waviness of jet streams.

The consequences of the theory can be examined in the framework of numerical simulations of the primitive equations with simplified physical parameterizations (32, 33), which are used in studies of general circulation (34, 35), the global hydrological cycle (36, 37), and extratropical large-scale atmospheric dynamics (38–40). A detailed description of the model setup is given in *SI Appendix*. We consider two contrasting cases in terms of the magnitude of the zonal mean wind with wavenumber 2 surface thermal forcing (*SI Appendix*, Fig. S3). Even with other factors, such as the vertical shear of the zonal mean wind, turbulent synoptic eddies, and large-scale latent heat, the large-scale atmospheric response to the surface thermal forcing is consistent with the solution of the planetary geostrophic motion described by the theory described here and by scaling analysis (20) (*SI Appendix*, Figs. S3 and S4). In particular, the response with weaker zonal mean wind is clearly larger than that with stronger zonal mean wind (*SI Appendix*, Fig. S3). Thus, the numerical simulations confirm that the theoretical solution describes a leading-order physics of the full nonlinear primitive equations.

## Discussion

### The Zonal Mean Wind, Vertical Stability, and *β*-Effect.

It is clear from our analysis that the magnitude of the zonal mean wind, *U*, underlies the response of stationary planetary-scale motion driven by surface thermal forcing. Essential here is that our characterization of strong versus weak zonal mean wind concerns the threshold $U_{\text{th}}\equiv 4H^{2}\beta_{L}S$, characterizing the strength of the vertical stability modulo the *β*-effect. Independent of whether we treat the system as having a rigid lid or being semi-infinite, when $U<U_{\text{th}}$ ($U>U_{\text{th}}$), the response of the system is sinusoidal (exponentially decaying), thereby demarcating vertical propagation from surface confinement. Therefore, when $U_{\text{th}}$ is large, the internal modes of the system reflect propagation of surface forcing and hence wavier jet streams. As the vertical stability increases, reflected by increasing *S* and *H*, themselves influenced by radiative–convective dynamics, modulated by latitude through the *β*-effect, the subthreshold waviness of jet streams is favored. Moreover, the pressure field $\phi_{L}$ is proportional to ${\left[U\left(k_{s}^{2}+\frac{1}{H}k_{s}\right)+\beta_{L}S\right]}^{-1}$ and hence is also enhanced as the zonal mean wind weakens. Clearly, although *U* depends on latitude *y* and height *z*, we have treated it as a representative constant in this analysis. The essential point is the dramatic change of the dynamics with $U_{\text{th}}$, and hence the sensitivity of its constituents when global forcing changes is the consequence of interest. For example, given the thermal wind balance, which relates the meridional temperature gradient to the vertical shear of the zonal mean wind, the magnitude of *U* is roughly proportional to the temperature difference between low and high latitudes. Along with this effect, there will be a secular change in vertical stability. Therefore, the slow variation in the global temperature distribution under a warming climate can be reflected in the planetary response we predict here.

### Arctic Amplification and the Midlatitude Westerlies.

The larger increase in high-latitude versus low-latitude temperatures under global warming is referred to as “polar amplification” and hence Arctic amplification in the Northern Hemisphere (41). The major planetary influence is a weakening of the meridional temperature gradient and hence a weakening of the zonal mean wind, argued to be responsible for anomalous jet stream meandering, driving extreme flooding and drought in the midlatitudes (3, 4, 42). The natural question is, By what mechanism is the weakening of the zonal mean wind linked to the meandering of jets?

The conditions for baroclinic instability of a linearized planetary geostrophic equation have recently been addressed in the framework of a two-layered model (21). In contrast to traditional baroclinic instability on the synoptic scale, which requires a vertical shear larger than a threshold (13), baroclinic instability on the planetary scale occurs when the vertical shear is smaller than a threshold. Moreover, there exists an optimal vertical shear for the maximum growth of a given perturbation.* Finally, consistent with the theory, using the numerical simulations described in *SI Appendix*, we find a nonlinear increase in the stream function magnitude as the midlatitude zonal mean wind decreases below a threshold of ~10 m/s.

Here we have focused on the fact that, due to land–ocean contrast and its seasonality, the Earth is not zonally symmetric, thereby significantly influencing the barotropic mean westerly winds. In consequence, we have treated the response of the mean field to surface thermal forcing quantitatively, using planetary geostrophic motion. We found that conditions under which $U<U_{\text{th}}$ lead to much wavier mean westerlies in midlatitudes. Such conditions are consistent with Arctic amplification due to the reduction in the mean wind accompanying the reduction in meridional temperature difference.

### Extreme Summer Weather.

Midlatitude extreme weather events are of contemporary interest and can be understood by assessing the change in the mean fields. For example, relative to winter, the mean summer westerlies are weaker and thereby sensitive to zonally asymmetric thermal forcing. Moreover, observations, modeling, and paleo-proxies (43–47) indicate a decrease in synoptic variability with weakened westerlies under global warming. Thus, in the absence of countervailing processes intensifying the westerlies, such as upper-level tropical warming, wavier summer jet streams will be more common. Both the theory described here and quasi-stationary resonant synoptic-scale waves (2) lead to the same qualitative conclusion.

### Further Issues.ss

There are many challenges associated with understanding how global warming influences midlatitude jet structure. Although Arctic amplification reduces the meridional temperature gradient in lower atmospheric levels (3), upper tropospheric tropical warming increases the temperature gradient in upper atmospheric levels (48). These competing factors compromise our ability to ascribe a single cause to a decrease in the zonal mean zonal wind (49).

We calculated the response of linearized planetary geostrophic motion to zonally asymmetric thermal forcing. However, if the meridional wind is comparable in magnitude to the zonal mean wind, one must consider nonlinear advection terms in the analysis. Moreover, one should examine how synoptic eddies grow from nonzonal mean winds and then decay to impact the planetary-scale mean field. This form of wave–mean-field interaction could differ from that with a zonally symmetric mean field.

Although our theoretical predictions might be found in climate model simulations, the Coupled Model Intercomparison Project 5 models exhibit systematic hemispheric historical biases in extratropical cyclones (50) and give a poor consensus on blocking projections (51). Increased model resolution and improved parameterizations may reduce such biases (52, 53), indicating that future models may provide test beds for the basic mechanisms proposed here.

## Conclusion

Wave–mean-flow interactions constitute a central process shaping the low-frequency dynamics of the large-scale atmosphere. The zonal mean field is typically considered because its zonally symmetric part reflects the dominance of the meridional temperature gradient. Therefore, this choice does not reflect the influences of zonally asymmetric thermal forcing. However, treating the dynamics within a multiscale formalism allows the mean field to be the solution of the planetary geostrophic equations. This shows the mutual interaction between the planetary and synoptic scales, providing a general framework for wave–mean-field interactions on the largest scales.

We emphasize that although we have not considered the influence of synoptic-scale eddies, they can be incorporated in our general treatment of the planetary heat transport equation. Although traditional baroclinic instability is based on a zonally symmetric flow, as shown here zonally asymmetric thermal forcing induces wavier jets and hence one does not expect similar behavior of synoptic eddies from the same forcing. Indeed, while other nonzonal mean flows may underlie baroclinic instability on the synoptic scale, in the context considered here the synoptic eddies could influence the mean field on the planetary scale. Despite this phenomenon being naturally incorporated into our framework, the associated complexities cloud our central message.

Finally, extreme weather events in the midlatitudes are associated with wavier jet streams and appear to be more frequent. The wavier jets are correlated with Arctic amplification, which, due to the reduced meridional temperature gradient, weakens zonal flow. Here we have provided a dynamical mechanism for this coupling by quantitatively examining the influence of zonally asymmetric thermal forcing due to land–ocean contrast. We find that weaker zonal mean flow amplifies the meridional response of the pressure and wind fields. Therefore, as Arctic amplification further weakens the zonal flow, one expects the asymmetric forcing to further enhance jet undulations and thus the meridional localization of transport.

## Supplementary Material

Supplementary File

## Data Availability

All study data are included in the article and/or *SI Appendix*.

## Footnotes

^*^Note that the threshold for the planetary-scale baroclinic instability is proportional to *β_LSH_* and the condition $U<\frac{1}{8}\beta_{L}SH$, where *U* is the barotropic mean midlatitude wind, must be satisfied.
