# Supplementary material for: Wavier jet streams driven by zonally asymmetric surface thermal forcing
Source: Proc Natl Acad Sci U S A. 2022 Sep 12;119(38):e2200890119. doi: 10.1073/pnas.2200890119 (PMC9499547; doi:10.1073/pnas.2200890119)
Supplement: Supplementary File [file pnas.2200890119.sapp.pdf]

Supporting Information Text

A. Tests of the theory using a Gray-Radiation Aqua-Planet Moist General Circulation Model (GCM). The theoretical predictions are based on planetary geostrophic motion driven by thermal forcing confined to the Earth's surface with a barotropic mean wind. The theory does not consider the vertical shear of the mean wind field and synoptic eddies generated by baroclinic instability, which decrease the temperature gradient. The propagation of Rossby waves has been studied in the large-scale response of the atmosphere to thermal and/or orographic forcing with a barotropic mean wind (1, 2). The robustness of the idealized treatment can be tested in a realistic baroclinic atmosphere (3), where the overall results due to the baroclinicity do not change significantly.

A.1. Model Description. The numerical model used in the test is an idealized moist general circulation model, which is an extension of the dry model (4) that includes the influence of latent heat flux. The dry model is based on Newtonian cooling to a radiative equilibrium temperature and Rayleigh damping for the surface boundary layer. The radiative-convective process and boundary layer dynamics are indirectly represented, which differs from comprehensive GCMs. However, model used here overcomes these issues by implementing an explicit boundary layer and adopting a simplified gray radiative model to directly calculate the upward and downward fluxes rather than using Newtonian cooling. The surface is a slab of water with a specific heat capacity and no horizontal transport. The large-scale convection is parameterized by the Betts-Miller scheme. The primitive equations are solved numerically using a standard Eulerian spectral dynamical core with triangular truncation. Time integration uses a leapfrog scheme with a Robert filter and a fourth-order hyperdiffusion stabilization method. A more detailed description of the model is given in (5, 6).

A.2. Asymmetric surface heat flux and surface temperature forcing. To control the overall magnitude of the zonal-mean zonal wind we impose an additional asymmetric surface heat flux as

$$Q = A\Phi_1(\theta) + B\Phi_2(\theta)\Phi_3(\theta)\sin(4\pi\phi), \qquad [1]$$

where

$$\begin{aligned}\Phi_1(\theta) &= \frac{1}{2}[1+\tanh(\alpha[\theta-30^\circ])]\\ \Phi_2(\theta) &= \frac{1}{2}[1+\tanh(\alpha[\theta-20^\circ])]\\ \Phi_3(\theta) &= \frac{1}{2}[1+\tanh(\alpha[60^\circ-\theta])]. \end{aligned} \qquad [2]$$

Here, θ is the latitude, ϕ the longitude, and α is a constant controlling the shape of the latitudinal functions. The first term, $A\Phi_1(\theta)$, represents an additional zonally symmetric surface heat flux at higher latitudes (above 30°). This controls the overall magnitude of the mean zonal wind, including the vertical shear. When A increases (decreases), the additional positive (negative) heat flux at high latitudes reduces the meridional temperature gradient, which decreases (increases) the overall magnitude of the mean zonal wind. The zonally asymmetric thermal forcing is $B\Phi_2(\theta)\Phi_3(\theta)\sin(4\pi\phi)$, where $\Phi_2(\theta)$ and $\Phi_3(\theta)$ act to confine the zonally asymmetric forcing between $20^\circ N$ and $60^\circ N$.

Figure S1 shows the default surface heat flux Q. Two cases are considered for comparison. The strong Arctic amplification case ($A = 100\ W/m^2$) is shown in (a) and a cold Arctic case ($A = -50\ W/m^2$) is shown in (b). The zonally asymmetric surface heat flux is obtained by subtracting the zonal average of Q from (a) and (b), as shown in (c) and (d) respectively. The zonally asymmetric thermal heat flux is given as a wavenumber 2 pattern and is exactly same for the both cases. This surface heat flux is applied as stationary thermal forcing during the entire simulation.

The central control of the surface temperature is local radiative-convective equilibrium. Therefore, we expect the temperature to exhibit a similar spatial pattern to that of the surface heat flux Q. Figure S2 shows the time average of the surface temperature for the two cases described in Figure S1; (a) Arctic amplification ($A = 100\ W/m^2$) and (b) a cold Arctic ($A = -50\ W/m^2$). The surface temperature at low latitudes is approximately $30^\circ C$. In case (a), the surface temperature in the Arctic basin is approximately $5^\circ C$. On the other hand the cold Arctic case in (b) has a surface temperature of approximately $-35^\circ C$ and hence has a larger meridional temperature gradient. Clearly, in the Arctic amplification case higher latitudes are warmer and the meridional temperature gradient decreases. After subtracting the zonal average from (a) and (b), the zonally asymmetric component of the temperature in Figures S2 (c) and (d) exhibit the spatial pattern of the surface heat flux shown in Figures S1 (c) and (d). Even accounting for the slight difference in the position of the maximum and the shape of the local pattern, the phase of the surface temperature is nearly the same as that of the surface heat flux Q.

A.3. Model simulations. We treat two contrasting cases distinguished by the magnitude of the zonal-mean zonal wind, controlled by the zonally symmetric surface heat flux. We impose the same zonally asymmetric surface thermal forcing (wavenumber 2) and show the weak and strong zonal-mean zonal wind cases in Figures S3 (a) and (b) respectively. The thermal wind balance shows that the Arctic warming in (a) leads to a decrease in the zonal-mean zonal wind, which is approximately 15 m/s near the tropopause. In contrast, the strong zonal-mean zonal wind case in (b) gives a value of approximately 30 m/s. As shown in the stream function at 300 mb (Figs. S3 (c, d)) and at 850 mb (Figs. S3(e, f)), the response of the weak zonal-mean zonal wind field to the zonally asymmetric forcing is larger than that of the strong case. This is the same as in the theoretical calculation described in the main text. In particular, the $\pi/2$ phase difference between the upper and lower atmosphere calculated in

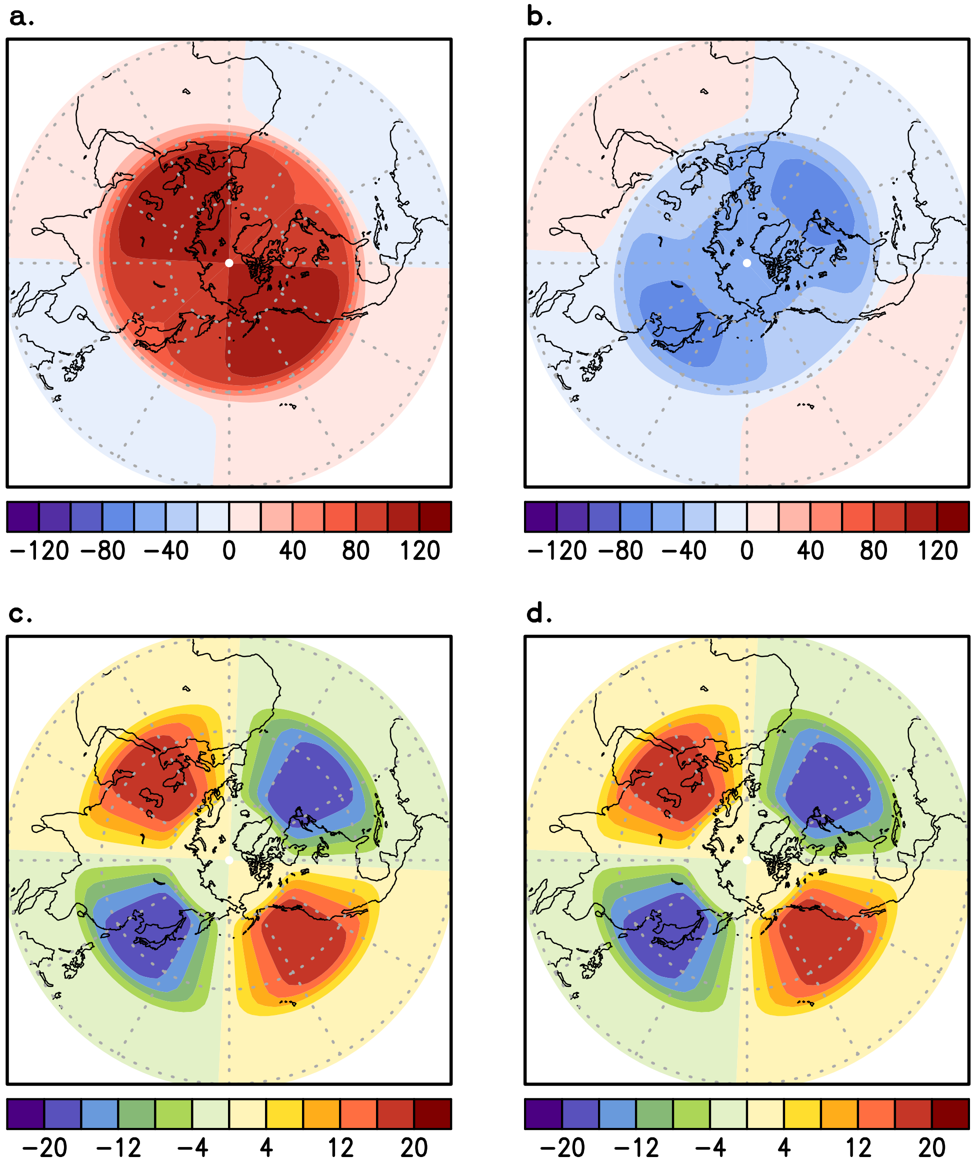

Fig. S1. The imposed surface heat flux Q when A is equal to (a) $100\ W/m^2$ and (b) $-50\ W/m^2$. The zonally asymmetric contributions are obtained by subtracting the zonal average of Q, and are shown in (c) and (d) respectively. These latter two are equivalent to $B\Phi_2(\theta)\Phi_3(\theta)\sin(4\pi\phi)$.

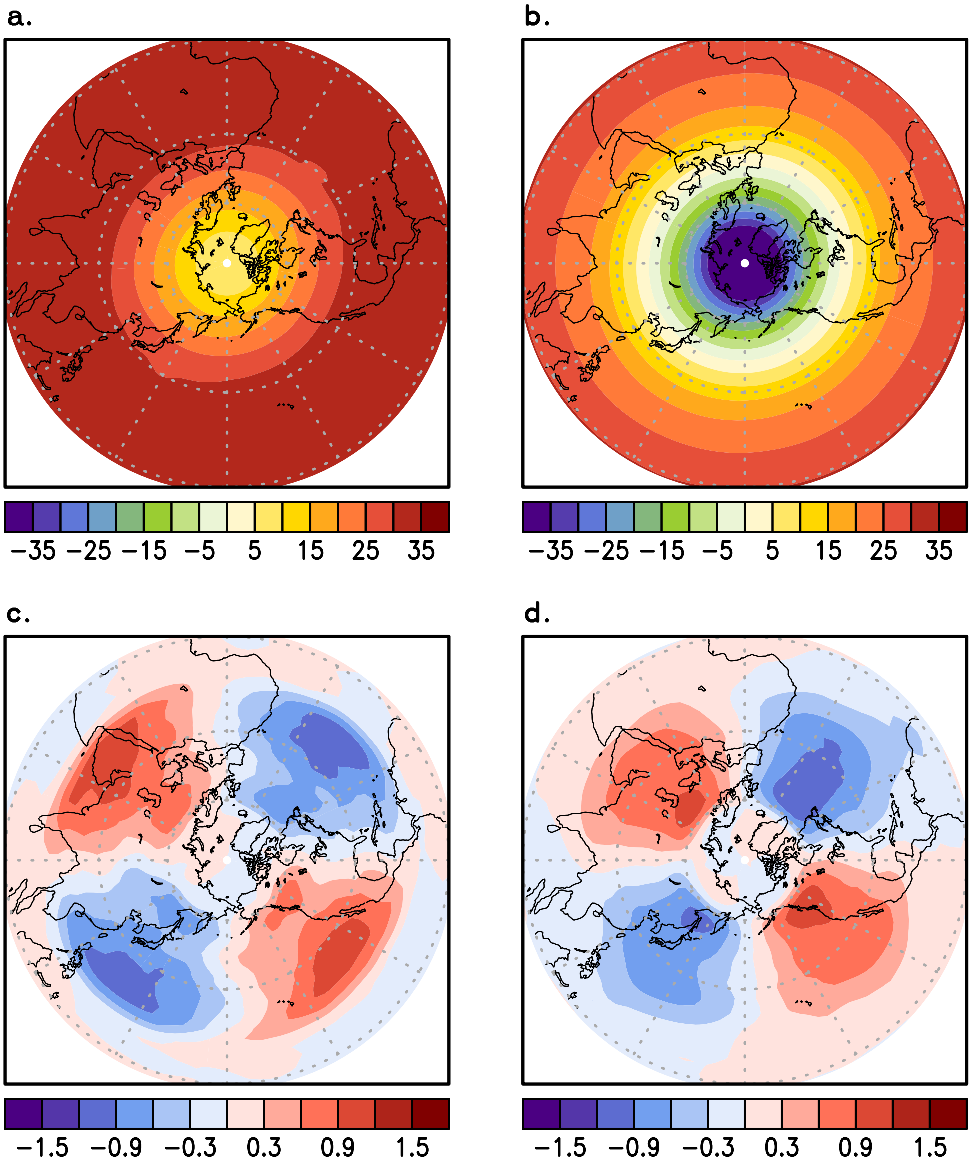

Fig. S2. The time-averaged surface temperature when A is equal to (a) $100\ W/m^2$ and (b) $-50\ W/m^2$. The zonally asymmetric contributions are obtained by subtracting the zonally and time averaged temperature, and are shown in (c) and (d) respectively.

the theory are also found in the numerical simulations. Furthermore, the spatial pattern of the meridional velocity v and the vertical velocity ω shown in figure S5 show that the Sverdrup relation is satisfied, and hence that the leading order dynamics is that of planetary geostrophic motion.

Note that because of the use of the pressure coordinates the usual vertical component of the velocity w is replaced by ω (usually reserved for vorticity in fluid mechanics), such that $\partial\omega/\partial P = \partial w/\partial z$, so that positive ω represents downward flow.

A.4. Sverdrup relation and planetary scale geostrophic motion. Because the numerical model is based on the primitive equations and the theory uses an asymptotic limit of the primitive equations, we use the former as a test of the latter. The essential part of planetary geostrophic motion is the Sverdrup relation, which arises from the continuity equation combined with the leading order β-effect. This distinguishes planetary geostrophic motion from quasi-geostrophic motion in which the β-effect comes in at $O(\epsilon)$.

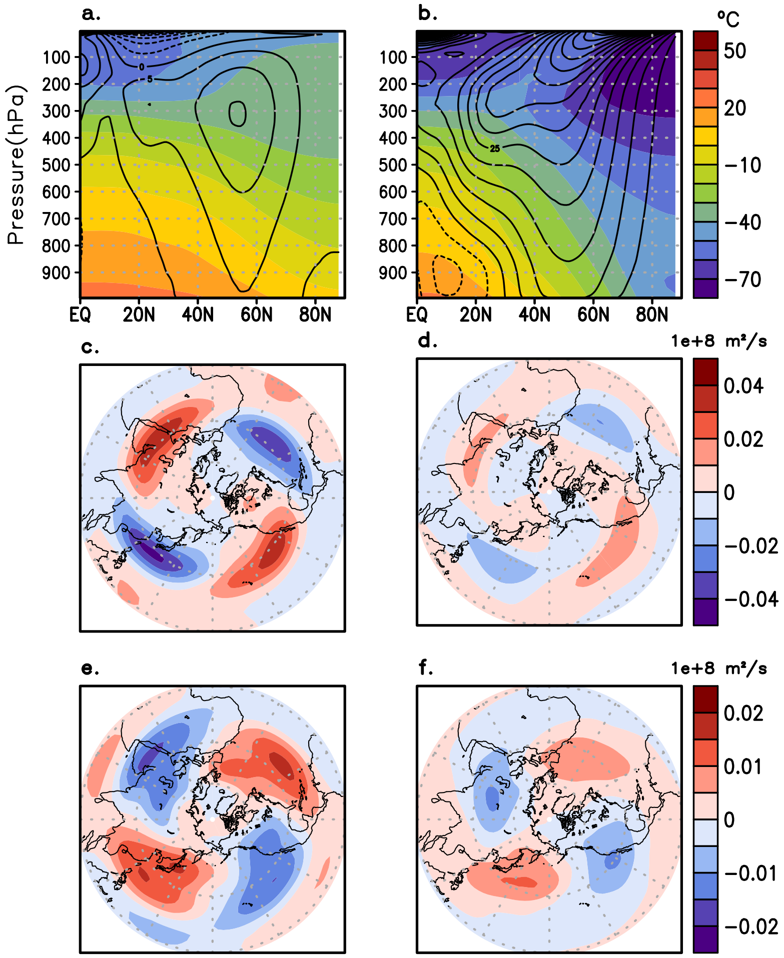

Fig. S3. The large-scale response of the atmosphere to surface thermal forcing in an idealized global climate model. Weak zonal-mean zonal wind generated by increased thermal forcing (a) and strong zonal-mean zonal wind generated by cooling (b) in high latitudes. The contour shows zonal-mean zonal wind (m/s) and the color shows the temperature ($^\circ C$). We compare the stream functions at 300 mb in the weak case (c) and in the strong case (d), and similarly at 850 mb for the weak case (e) and the strong case (f).

Figure S4 shows the meridional velocity v and the vertical velocity ω for the weak (a, c, e) and strong (b, d, f) zonal-mean zonal wind cases. The key observation is that whereas the strong wind case does not show organized meridional or vertical velocities, the weak wind case clearly does, with the same wavenumber 2 horizontal structure discussed above. The positive (negative) vertical velocity ω at 500 mb lies between the positive (negative) meridional velocity v at 300 mb and the negative (positive) one at 850 mb. Because the vertical velocity nearly vanishes at the surface and near the tropopause, the structure of v and ω shown in the simulations shown here is consistent with the Sverdrup relation, which in dimensional form is $f\partial\omega/\partial P = \beta v$, where f is the Coriolis parameter. Therefore, planetary geostrophic motion describes the leading-order dynamics and our theory provides a quantitative approximate dynamical framework.

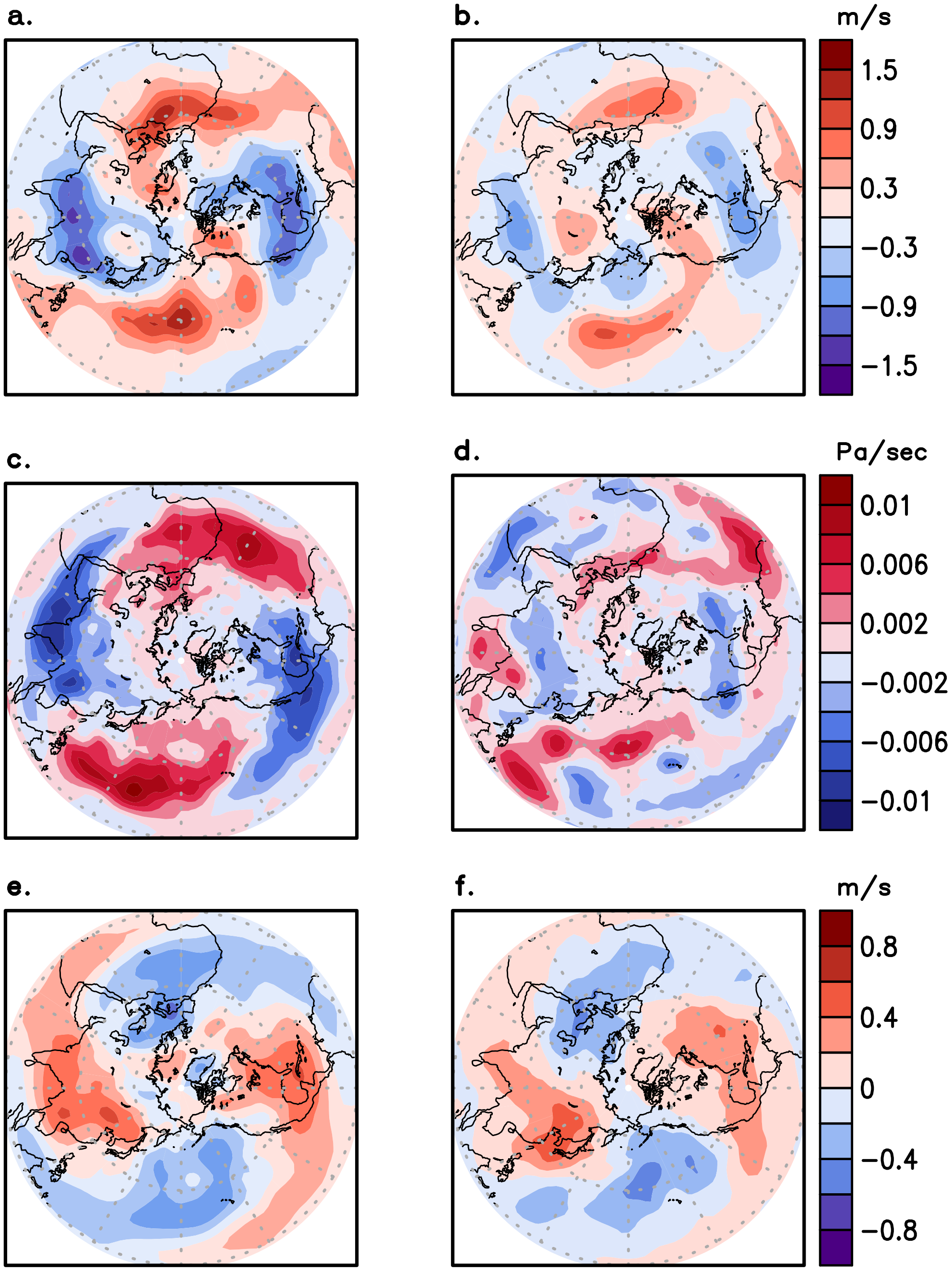

Fig. S4. Meridional velocity v at 300 mb (a) and 850 mb (e), and the vertical velocity ω at 500 mb (c) for the weak zonal-mean zonal wind case. The same quantities for the strong zonal-mean zonal wind case are shown in (b), (d), and (f).
